# ReadChop: a high-performance demultiplexer for long-read sequencing data

**DOI:** 10.1093/bioinformatics/btag339

**Published:** 2026-06-25

**Authors:** Chen Jiang, Yuanyan Xiong

**Affiliations:** Department of Biochemistry, Key Laboratory of Gene Engineering of the Ministry of Education, School of Life Sciences, Sun Yat-sen University, Guangzhou, 510275, China; Department of Biochemistry, Key Laboratory of Gene Engineering of the Ministry of Education, School of Life Sciences, Sun Yat-sen University, Guangzhou, 510275, China

## Abstract

**Summary:**

Long-read sequencing (LRS) platforms offer extended read lengths but present computational challenges due to high error rates and frequent insertion-deletion (indel) artifacts. While sample multiplexing is essential for cost-efficiency, existing demultiplexing solutions face a dichotomy: vendor-provided tools (e.g., Dorado) often lack the structural flexibility required for highly non-canonical designs, while open-source tools (e.g., Cutadapt) often lack the speed or algorithmic robustness to handle custom, high-complexity barcode designs. Here, we present ReadChop, a high-performance demultiplexer implemented in Rust. ReadChop leverages Myers’ bit-parallel algorithm to efficiently model indel-rich error profiles and employs a streaming architecture to ensure low memory footprint. Benchmarking demonstrates that ReadChop achieves classification precision exceeding 99.99% on both simulated datasets—even under ultra-high multiplexing conditions (e.g., 13 824-plex)—and empirical SARS-CoV-2 amplicons. Furthermore, it excels in filtering in silico chimeras (0.1% miss rate) and exhibits linear computational scalability on ultra-long templates (up to 100 kb). Crucially, it significantly accelerates execution speeds—being >6 times faster than Dorado, >2 times faster than Nanoplexer, and >30 times faster than Cutadapt—with memory usage consistently below 200 MB. ReadChop provides a flexible, robust solution for processing massive LRS datasets with non-canonical experimental designs.

**Availability and Implementation:**

Source code and documentation are freely available under the MIT license at https://github.com/cherryamme/ReadChop.

## 1 Introduction

Long-read sequencing (LRS) technologies, specifically Single Molecule Real-Time (SMRT) sequencing by Pacific Biosciences and nanopore sequencing by Oxford Nanopore Technologies (ONT), have fundamentally advanced genomics. Unlike traditional short-read platforms (e.g., Illumina, typically <300 bp), LRS generates continuous reads typically ranging from kilobases (kb) to hundreds of kilobases, occasionally reaching the megabase (Mb) scale. This capability enables the resolution of complex genomic regions, including repetitive elements and structural variants >50 bp (‘Nanopore Sequencing’ 2024; Logsdon *et al*. 2020). However, the adoption of LRS is frequently constrained by computational bottlenecks in data preprocessing (Wang *et al.* 2021). While recent major upgrades in hardware and chemistry—such as Oxford Nanopore’s shift to R10.4.1—have achieved single-read accuracies exceeding 99% (Kim *et al.* 2024), these reads maintain a distinct error profile compared to short-read data, characterized by a specific propensity for insertion-deletion (indel) artifacts. Additionally, artifacts such as chimeric reads—resulting from template switching or concatenation during library preparation—complicate downstream analysis (Lasken and Stockwell 2007, White *et al.* 2017, Lu *et al.* 2023, Heinz *et al.* 2026).

To maximize sequencing efficiency, libraries are routinely constructed with synthetic sequences, including adapters, barcodes, and Unique Molecular Identifiers (UMIs). While short-read platforms require compact designs due to length constraints (Al Bkhetan and Wang 2024, Sullivan and Pachter 2024, Fu *et al.* 2025), the extended read length of LRS accommodates longer, more complex barcode architectures. This flexibility significantly enhances sample multiplexing throughput and scalability, making large-scale sequencing runs increasingly cost-effective (Srivathsan *et al.* 2018, Stephenson and Nivala 2023, Hebert *et al.* 2025, Van Der Toorn *et al.* 2025a).

Despite these advantages, effective demultiplexing remains a critical challenge. While vendor-provided solutions like ONT Dorado have recently introduced support for custom barcode sequences, they fundamentally operate under the structural assumptions of standard library kits, lacking the architectural flexibility required for non-canonical, multi-segment, or highly customized indexing schemes (Qi *et al.* 2024, Van Der Toorn *et al.* 2025a). Conversely, general-purpose tools like Cutadapt and fastp were originally engineered for short-read data (Martin 2011, Cheng *et al.* 2024, Sullivan and Pachter 2024, Fu *et al.* 2025), and newer long-read specific tools such as fastplong lack demultiplexing capabilities altogether. When applied to LRS, these tools often demonstrate limited efficiency and accuracy, primarily due to high computational costs and an inability to adequately model indel-rich error profiles. While Nanoplexer achieves significantly higher processing speeds, it is limited by reduced flexibility and memory management issues. Furthermore, other approaches remain restricted to specialized applications, such as single-cell RNA-seq and tRNA-seq (Han *et al.* 2023, Papetti *et al.* 2023, Cheng *et al.* 2024, van der Toorn *et al.* 2025b).

To address these limitations, we present ReadChop, a high-performance toolkit designed for the rapid classification and demultiplexing of LRS reads. Built upon Myers’ bit-parallel algorithm (Myers 1999, Šošić and Šikić 2017), ReadChop is implemented in Rust to ensure memory safety and computational efficiency. It supports multi-threaded parallel processing and complex multi-pattern recognition, while explicitly incorporating mechanisms for filtering chimeric reads and a web-based interface for custom library configuration. Furthermore, its streaming architecture prevents memory overflow when handling massive datasets, offering a robust solution for high-throughput LRS analysis.

## 2 Methods

### 2.1 Data sources

We evaluated ReadChop using both simulated and empirical datasets. Simulated reads were generated using Badread with the Nanopore 2023 error model to replicate the error characteristics of current platforms, enabling the precise calculation of read-level demultiplexing accuracy. For real-world validation, we utilized two distinct empirical datasets to ensure robust evaluation across different library types. First, we used 50 SARS-CoV-2 samples from the public repository PRJNA675364 (Bull *et al.* 2020), which were constructed using the ONT Rapid Barcoding Kit (SQK-RBK004) and represent a standard long-amplicon workflow. Second, to evaluate performance on non-amplicon data, we incorporated a multispecies Whole Genome Sequencing (WGS) dataset (PRJNA1364846) prepared with the ONT Rapid Barcoding Kit V14 (SQK-RBK114).

### 2.2 Barcode identification algorithm

The core of ReadChop is a barcode recognition module based on Myers’ bit-parallel algorithm. This approach accelerates dynamic programming via bitwise operations, significantly reducing the computational cost of matching short patterns. Crucially, it maintains high sensitivity to indel errors while ensuring time complexity remains linear with respect to read length. Unlike tools relying on fixed-window alignment, ReadChop implements a flexible local scanning approach. Users can constrain search intervals for potential barcodes, optimizing the trade-off between identification flexibility and execution speed.

To accommodate complex library architectures, ReadChop supports dual barcoding and combinatorial barcoding (accommodating asymmetric barcode pairs on opposite ends of a read), alongside three-segment barcodes, reverse-complement patterns, and fuzzy positional matching. Furthermore, to address the lower sequence quality often observed at read ends in Nanopore sequencing, we utilize a hierarchical “anchor-based” recognition strategy. Specifically, the algorithm first locates the innermost barcode to serve as a positional anchor. Using these coordinates, ReadChop then defines the search intervals for the adjacent, outermost barcodes. This practical approach helps maintain demultiplexing robustness in noise-prone terminal regions and facilitates multi-level demultiplexing, allowing for the reuse of identical barcode sequences across different structural tiers.

### 2.3 Anomaly detection and filtering

Nanopore sequencing can produce artifacts such as short fragments and signal breaks. ReadChop permits the exclusion of reads below user-defined length thresholds. For fragmented or structurally incomplete reads, the software offers selectable single-end or dual-end matching modes. To detect chimeric reads, ReadChop performs multiple scanning rounds within a single read to identify repetitive adapters or inconsistent barcode structures, subsequently triggering filtration or splitting protocols based on user configuration.

### 2.4 Software architecture and implementation

ReadChop is implemented in Rust (Edition 2023), leveraging zero-cost abstractions and memory safety guarantees. The system follows a Producer-Consumer concurrency model, where tasks exchange data via bounded channels. All I/O operations utilize streaming execution to avoid loading entire datasets into memory. This design ensures the memory footprint remains low (typically hundreds of MB), even when processing tens of millions of reads.

### 2.5 Web configuration tool

To enhance usability, we developed the ReadChop Configuration Tool, a lightweight web application for visual parameter generation. It allows users to design barcode structures, define rule sets, and preview workflows without manually editing complex configuration files. Configurations can be exported directly for command-line execution, streamlining the transition from experimental design to data processing.

## 3 Results

### 3.1 Accuracy on complex barcode structures

**Figure 1:**
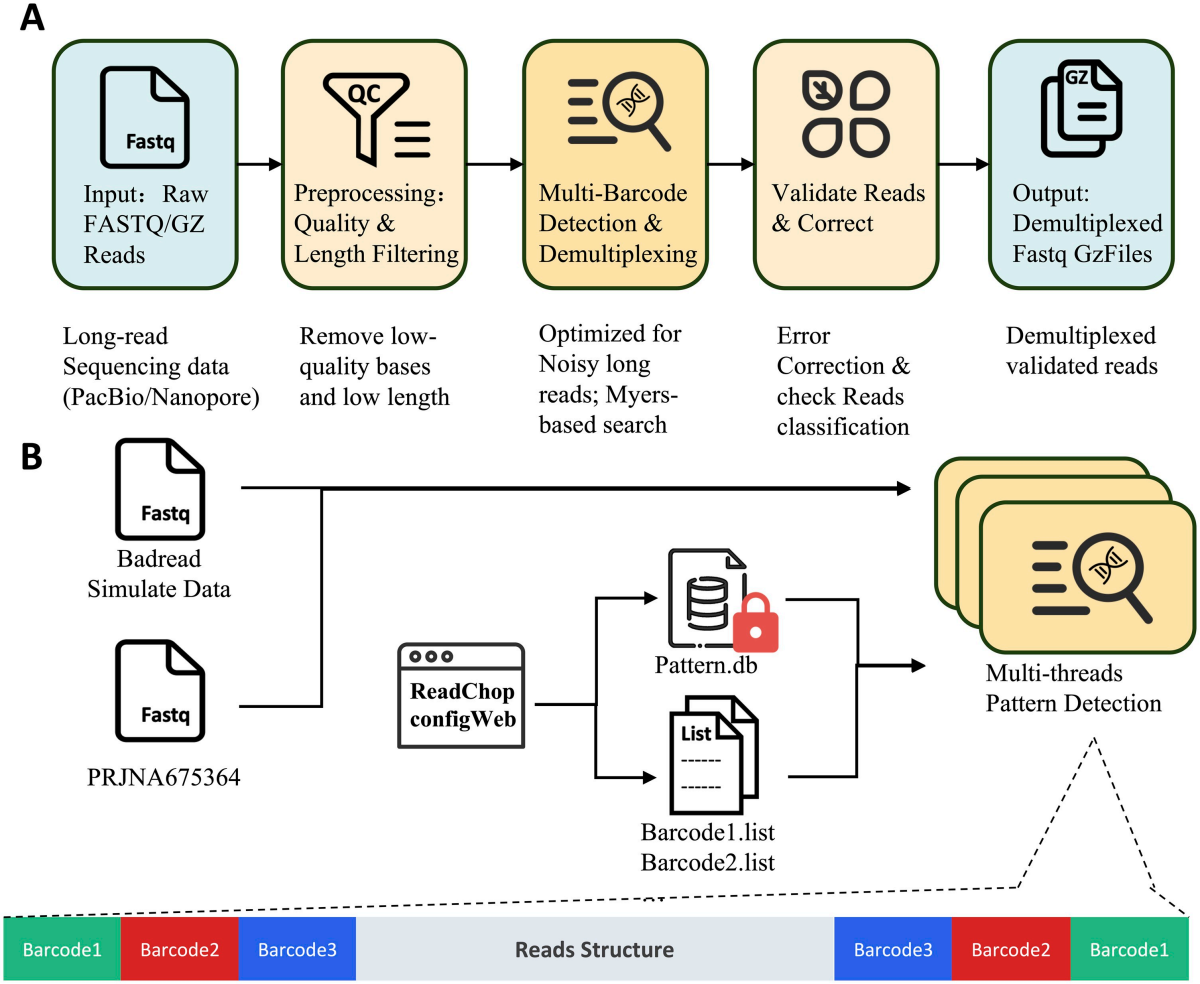
Overview of the ReadChop workflow and experimental design. (A) The core processing pipeline, ranging from raw FASTQ/GZ input and quality/length pre-filtering to multi-barcode detection based on Myers’ bit-parallel algorithm, read validation, and final demultiplexed output. (B) Data construction strategy for performance benchmarking. Simulated datasets featuring a complex three-segment barcode structure (Barcode1-Barcode2-Barcode3) were generated via Badread, while 50 empirical SARS-CoV-2 samples (PRJNA675364) were utilized for real-world validation. The schematic also illustrates the generation of the pattern database (Pattern.db) using the ReadChop ConfigWeb tool.

**Figure 2:**
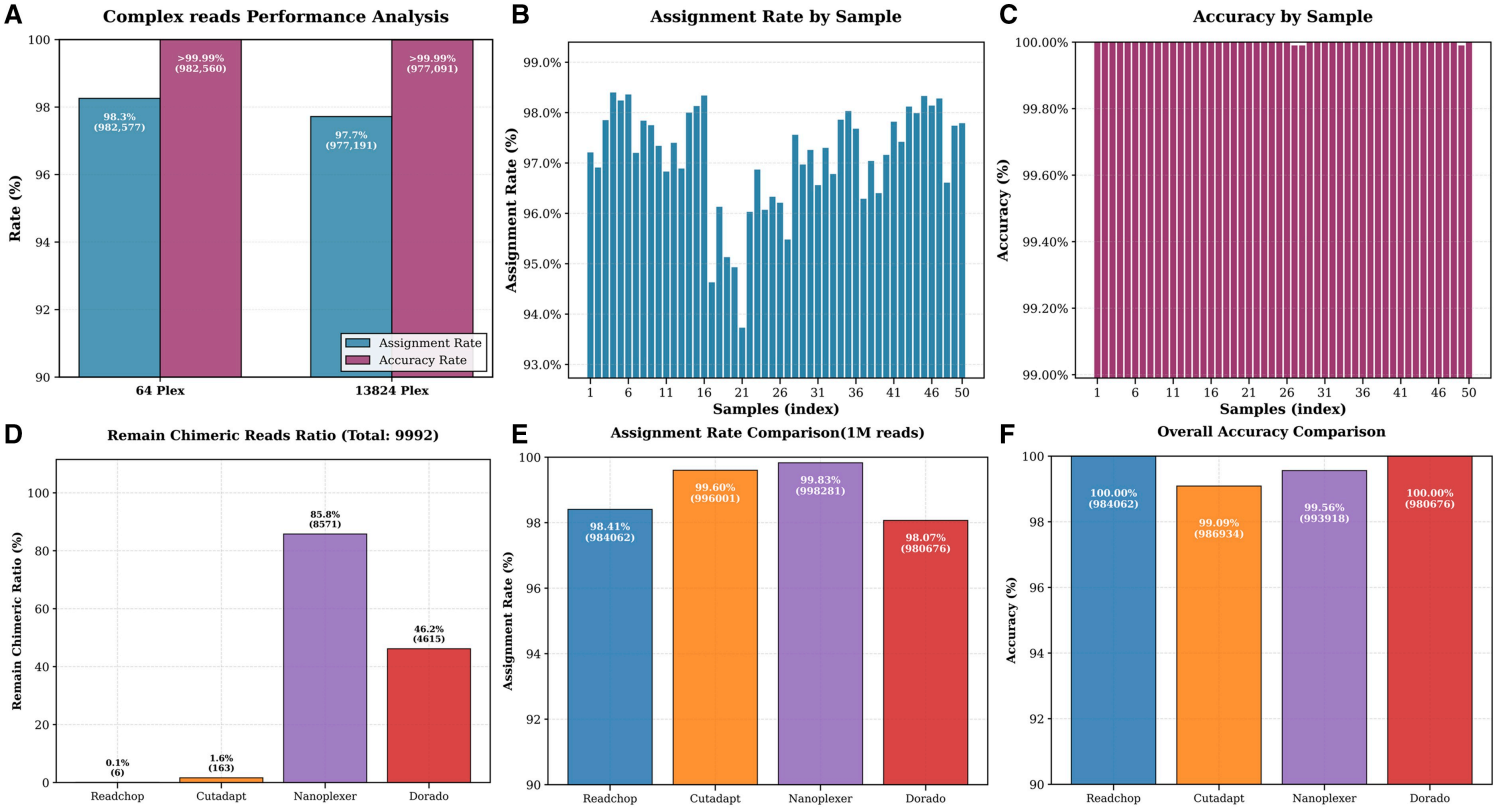
Assessment of demultiplexing accuracy and chimera detection on simulated and empirical datasets. (A) Assignment rate and classification accuracy evaluated on simulated datasets (106 reads) with complex three-segment barcode structures, demonstrating ReadChop’s robust performance across both standard (64-plex) and ultra-high multiplexing (13 824-plex) scenarios. (B-C) Performance across 50 empirical SARS-CoV-2 samples (PRJNA675364), showing the distribution of assignment rates and overall accuracy. (D-E) Benchmarking of ReadChop against Cutadapt, Nanoplexer, and Dorado on standard simulated datasets (106 reads) in terms of overall assignment rate (D) and classification accuracy (E). (F) Comparison of chimeric read detection efficacy based on a ground truth of 9,992 true in silico chimeras. The bar plot displays the ratio and absolute number of residual chimeric reads after processing. ReadChop missed only 6 reads (0.1%), significantly outperforming Cutadapt, Nanoplexer, and Dorado.

To assess ReadChop’s performance in complex scenarios, we simulated a dataset (10^^6^ reads) featuring a three-segment barcode structure (Fig. 1B) to emulate multi-level indexing strategies. This design initially included 64 combinations with errors introduced via Badread. Under default parameters, ReadChop achieved an assignment rate of 98.3%, with classification precision exceeding 99.99% among successfully demultiplexed reads (Fig. 2A). Furthermore, to test the absolute limits of the tool’s scalability, we evaluated ReadChop on an ultra-high multiplexing scenario comprising 13 824 unique combinations. Remarkably, ReadChop maintained an assignment rate of 97.7% and a classification precision still exceeding 99.99% (Fig. 2A). These results demonstrate ReadChop’s exceptional scalability and stability in handling high error rates and complex positional variations, and massive index combinations.

In the validation using empirical data, we first analyzed 50 SARS-CoV-2 amplicon samples (∼ 9.6 × 10^6^ reads), a dataset exhibiting high heterogeneity due to amplification bias and variable sequencing quality. ReadChop maintained assignment rates between 93% and 99% across samples (Fig. 2B), with demultiplexing accuracy consistently surpassing 99.99% (Fig. 2C). Furthermore, to evaluate performance on non-amplicon libraries, we processed a 26-sample multispecies Whole Genome Sequencing (WGS) dataset (PRJNA1364846). As demonstrated in Supplementary Figure S1, available as supplementary material at *Bioinformatics* online, ReadChop delivered highly stable performance on this WGS data, achieving assignment rates consistently above 97.5% and sample accuracies exceeding 98.5%. These findings collectively confirm the tool’s robustness in the presence of real-world sequencing noise across diverse library preparation workflows.

### 3.2 Benchmarking against existing tools

To evaluate performance against current community standards, we benchmarked ReadChop against Cutadapt, Nanoplexer and Dorado using a simulated library based on the ONT SQK-RBK004 kit (48 barcode sets, 10^6 reads, ∼ 5 kb template length) under identical computational resources. Before evaluating quantitative performance, we first compared the functional capabilities of these tools (Table 1). While all evaluated tools support basic custom barcode sequences, ReadChop uniquely accommodates highly complex architectures, such as multi-level indexing and specific chimera filtration mechanisms. Regarding input formats, while Dorado features native BAM support, ReadChop ensures seamless integration into existing analysis pipelines through highly efficient standard input (stdin) streaming.

**Table 1:** Functional comparison of ReadChop and baseline demultiplexing tools.

Feature	ReadChop	Dorado	Cutadapt	Nanoplexer
Custom Barcode Sequences	Yes	Yes	Yes	Yes
Combinatorial Dual Barcoding	Yes	No	Yes	Yes
Multi-level Indexing (>2 tiers)	Yes	No	No	No
Chimera Filtering	Yes	No	Yes	No
Input Format	FASTQ	HTS format	FASTQ	FASTQ
Native BAM Support	No	Yes	No	No

In terms of assignment rate, ReadChop (98.41%) was marginally lower than Cutadapt (99.60%) and Nanoplexer (99.83%), but outperformed Dorado (98.07%) (Fig. 2D). This slight variance results from ReadChop’s stricter filtering of anomalous structures, prioritizing the quality of downstream analysis over the retention of potentially erroneous reads.

However, ReadChop demonstrated a decisive advantage in classification precision, achieving an overall accuracy of 100.00%, compared to 99.09% for Cutadapt and 99.56% for Nanoplexer (Fig. 2E). While Dorado also achieved 100.00% accuracy, its lower overall assignment rate indicates a conservative but less sensitive classification approach compared to ReadChop. The drop in Cutadapt’s accuracy highlights the limitations of short-read algorithms when applied to indel-rich LRS data.

To evaluate chimeric read detection (Fig. 2F), in silico chimeras were introduced into the simulated dataset using Badread at a rate of 1%. Based on the read length distribution profile (Supplementary Figure S4, available as supplementary material at *Bioinformatics* online), a threshold of 8000 bp was selected to accurately distinguish true chimeric reads from normal fragments. Evaluating against this ground truth (a total of 9992 chimeric reads), ReadChop missed only 6 chimeric reads (0.1%), significantly outperforming Cutadapt (163 missed). Notably, Dorado failed to identify 4615 chimeric reads (46.2%), and Nanoplexer missed 8571 (85.8%). These substantial discrepancies are likely attributable to their lack of specific post-processing steps for chimera resolution. Conversely, ReadChop’s superior precision stems from its rigorous internal structure scanning.

### 3.3 Runtime performance and resource efficiency

**Figure 3:**
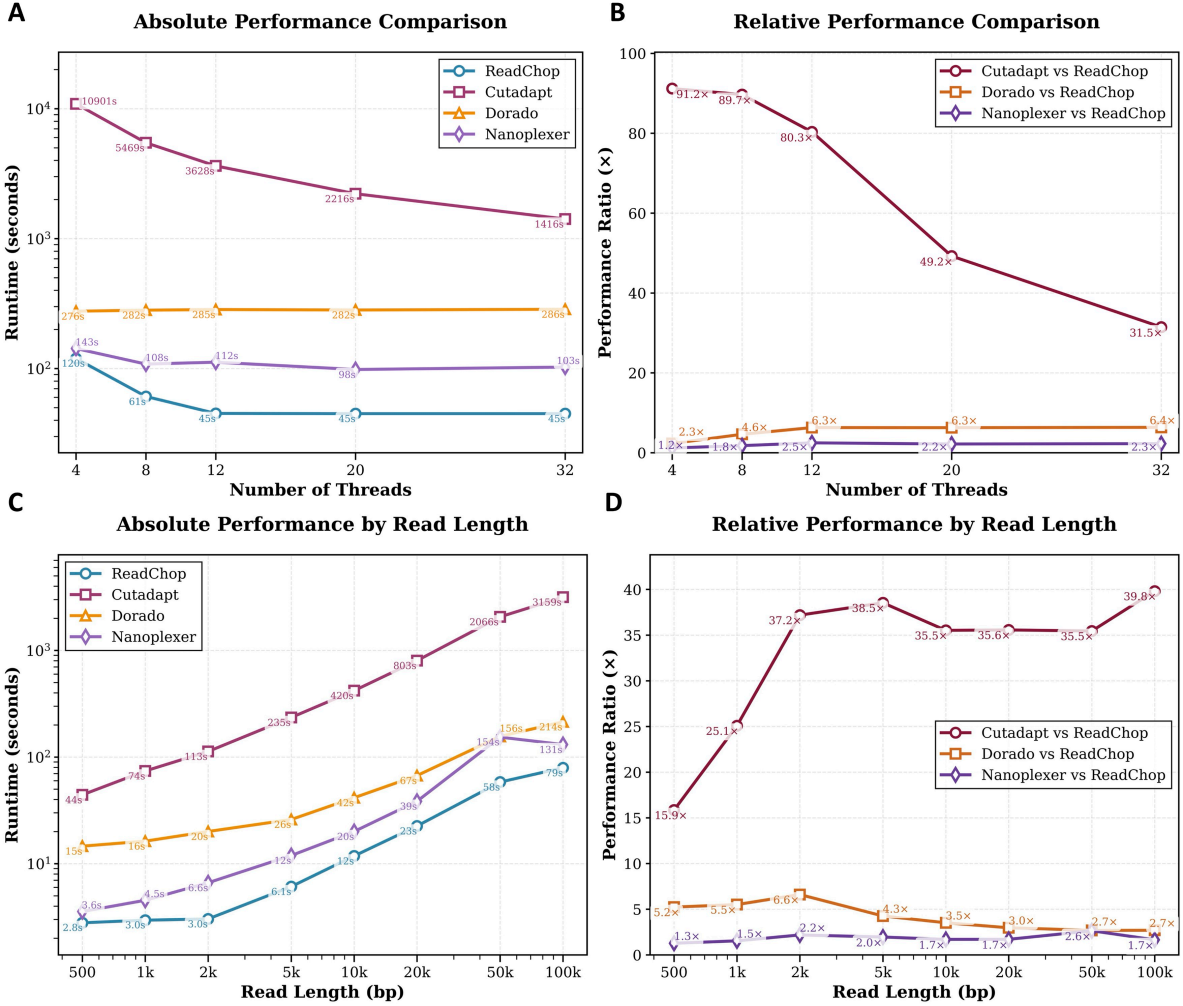
Runtime performance and scalability benchmarking. (A) Absolute runtime (seconds) of ReadChop and competing tools (Cutadapt, Dorado, Nanoplexer) across varying thread counts (4–32 threads) on a standard simulated dataset of 106 reads (∼5 kb length). (B) Relative performance ratio (speedup) of ReadChop compared to baseline tools across different thread configurations. (C) Computational scalability evaluated by absolute runtime across a broad range of simulated template lengths (500 bp to 100 kb) utilizing 20 threads. (D) Relative performance advantage of ReadChop against competing tools as a function of read length, demonstrating sustained efficiency on ultra-long sequencing data.

Performance benchmarking was conducted using hyperfine across varying thread counts. ReadChop demonstrated significantly superior processing speeds compared to competing tools. Notably, at thread counts exceeding 12, execution speed approached the disk I/O saturation point. While ReadChop consistently outperformed competing tools across all threading configurations, its advantage was most pronounced at optimal allocations (>12 threads), being >2 times faster than Nanoplexer, >6 times faster than Dorado, and >30 times faster than Cutadapt (Fig. 3A and B). Furthermore, to address the variable nature of LRS fragment lengths, we evaluated computational scalability across a broad range of template lengths (500 bp to 100 kb). As demonstrated in Fig. 3C and D, while the execution time of tools like Cutadapt scaled exponentially with longer reads, ReadChop maintained a distinct linear advantage, retaining robust processing speeds even on ultra-long templates (100 kb).

**Figure 4:**
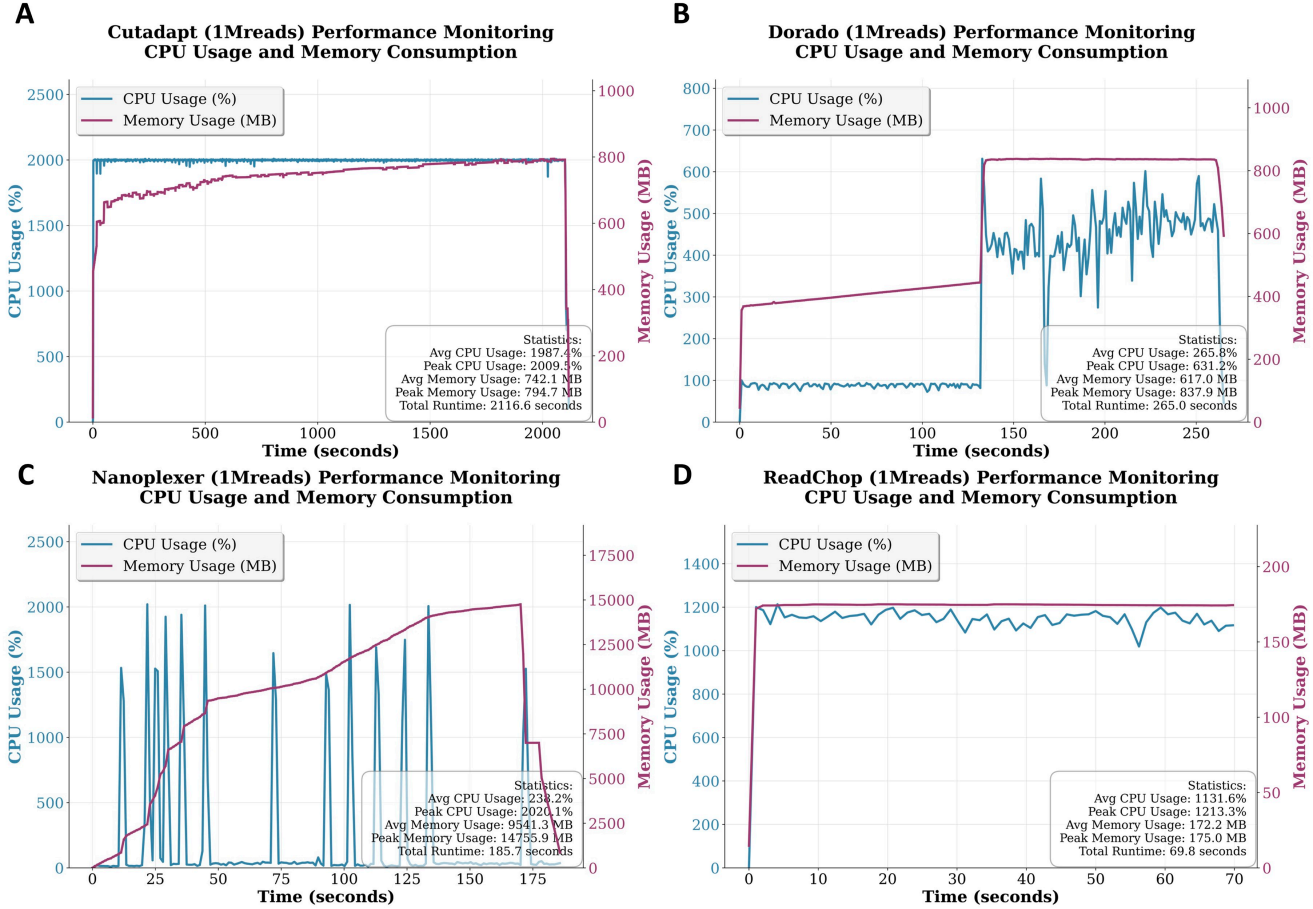
Real-time profiling of computational resource utilization. CPU usage (blue line, left *y*-axis) and memory consumption (red line, right *y*-axis) were monitored during the demultiplexing of a standard dataset (106 reads, ∼5 kb length) using 20 threads. Profiles are shown for (A) Cutadapt, (B) Dorado, (C) Nanoplexer, and (D) ReadChop. Notably, ReadChop maintained the lowest peak memory footprint (∼175.0 MB) and highly stable CPU allocation, whereas Nanoplexer exhibited severe memory overhead (peaking at ∼14.7 GB) and erratic CPU utilization.

In terms of resource efficiency, the Rust-based implementation of ReadChop ensured exceptional memory stability, peaking below 200 MB when processing 106 reads (Fig. 4A and D). In contrast, Nanoplexer, Cutadapt, and Dorado exhibited substantially higher memory footprints and suboptimal multi-threading scalability. Notably, even under stress testing with 107 reads (∼100 GB of data), ReadChop retained a minimal memory footprint and demonstrated linear scalability (Fig. S5), underscoring its suitability for ultra-large-scale sequencing initiatives.

## 4 Discussion

ReadChop was developed to address the specific preprocessing needs of long-read sequencing. As LRS applications expand into structural variant detection and metagenomic assembly, the characteristics of the data—high indel rates and barcode positional uncertainty—require specialized software solutions. Our results indicate that traditional tools rooted in short-read logic are often insufficient for these tasks, while standard vendor-provided tools may lack the flexibility required for highly customized designs.

Algorithmically, the deployment of Myers’ bit-parallel edit distance algorithm is central to ReadChop’s efficacy. Unlike Hamming distance approaches which assume mismatch-dominant errors, Myers’ algorithm intrinsically models indels. This is critical for LRS, where indels frequently cause positional offsets that lead traditional algorithms to misclassify reads. ReadChop maintains exceptional robustness against continuous or high-frequency indels, maximizing demultiplexing accuracy even in extreme scenarios involving ultra-high multiplexing (e.g., 13 824-plex configurations).

From a systems engineering perspective, the integration of a streaming architecture with Rust’s memory safety enables high-throughput processing on standard workstations. The benchmarking results illustrate a significant improvement in speed and resource efficiency compared to Cutadapt and Dorado, facilitating more efficient industrial-grade analysis pipelines.

While ReadChop demonstrates superior performance, opportunities for optimization remain. Current chimera detection relies primarily on structural features; future iterations could incorporate raw signal data to refine identification. Furthermore, recognizing the growing adoption of unaligned BAM formats in standard ONT workflows, introducing native BAM support is a priority for future releases to further streamline pipeline interoperability. Additionally, for extremely complex experimental designs, exploring inference strategies based on Graph-based models or Hidden Markov Models (HMM) represents a promising direction.

In conclusion, ReadChop provides a powerful, user-centric preprocessing utility for genomics research. By combining an algorithm optimized for LRS error profiles with an efficient implementation, ReadChop serves as a reliable bridge between raw experimental data and downstream biological insight.

## 5 Data and software availability

ReadChop is an open-source software distributed under the MIT license. The source code, documentation, and web configuration tool are freely accessible at GitHub (https://github.com/cherryamme/ReadChop). To ensure reproducibility, an archival snapshot of the code used for the experiments in this manuscript is available on Zenodo (https://doi.org/10.5281/zenodo.19851395). Furthermore, all scripts utilized for data simulation, metric computation, and baseline benchmarking are hosted in a dedicated reproducibility repository at https://github.com/cherryamme/ReadChop-manuscript. The empirical sequencing data used for validation are publicly available in the NCBI SRA under accession numbers PRJNA675364 and PRJNA1364846. The specific versions of all software and tools utilized are detailed in Supplementary Table S1, available as supplementary material at *Bioinformatics* online.

## Supplementary Material

btag339_Supplementary_Data
