# Supplementary material for: ReadChop: a high-performance demultiplexer for long-read sequencing data
Source: Bioinformatics. 2026 Jun 25;42(6):btag339. doi: 10.1093/bioinformatics/btag339 (PMC13310090; doi:10.1093/bioinformatics/btag339)
Supplement: btag339_Supplementary_Data [file btag339_supplementary_data.docx]

**Supplementary Table S1. Software tools, versions, and applications used in this study.**

| Software/Tool | Version | Application |
| --- | --- | --- |
| ReadChop | 1.0.2 | Proposed LRS demultiplexer |
| Dorado | 1.4.0+ba44a013 | Baseline demultiplexer |
| Cutadapt | 4.4 | Baseline demultiplexer |
| Nanoplexer | 0.1.2 | Baseline demultiplexer |
| Badread | 0.4.1 | Simulation of LRS data |
| Hyperfine | 1.19 | runtime benchmarking |
| psrecord | 1.4 | Real-time CPU and memory monitoring |
| SeqKit | 2.10.1 | FASTQ file manipulation and statistics |
| Seqtk | 1.5-r133 | FASTQ file formatting and subsampling |
| Python | 3.13.7 | Custom scripting and data visualization |


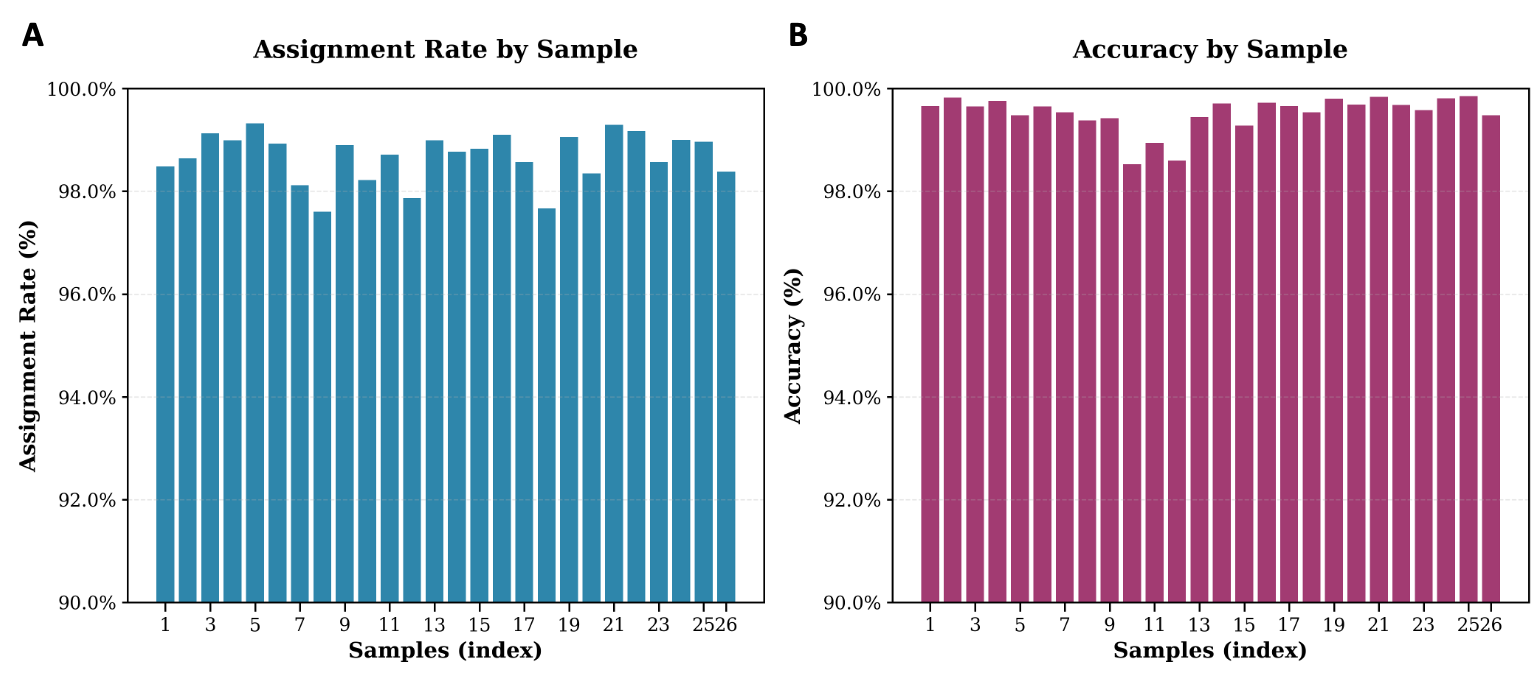


**Supplementary Figure S1. Demultiplexing performance on empirical Whole Genome Sequencing (WGS) data.** (A) Assignment rate and (B) classification accuracy across 26 non-amplicon multispecies WGS samples (PRJNA1364846) prepared with the ONT Rapid Barcoding Kit V14. The results demonstrate ReadChop's robust performance on highly heterogeneous, non-amplicon libraries, with assignment rates consistently above 97.5% and sample accuracies exceeding 98.5%.


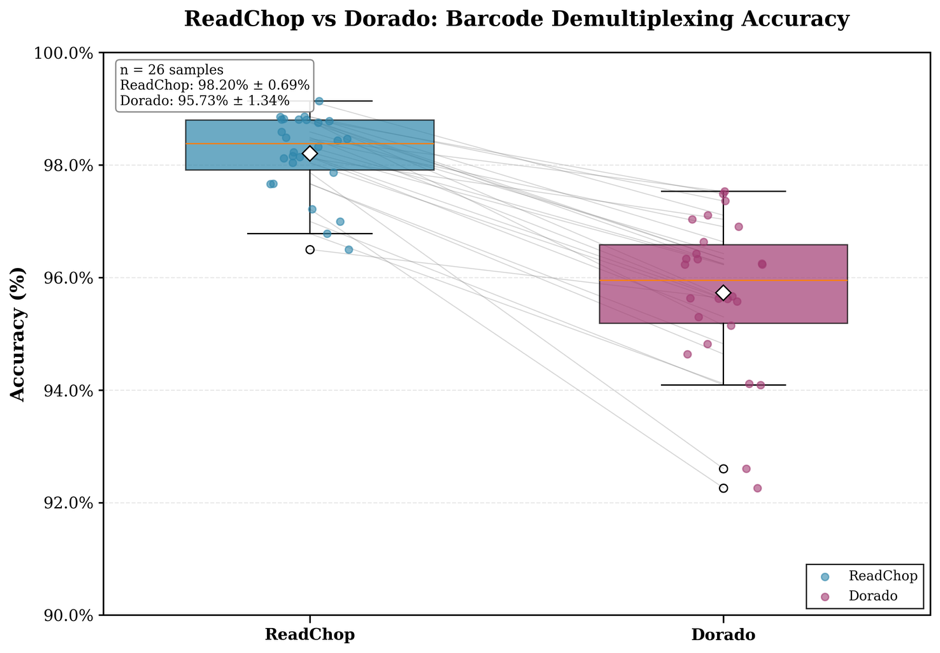


**Supplementary Figure S2. Comparative analysis of demultiplexing accuracy on empirical WGS data.** Boxplot comparing the classification accuracy of ReadChop and Dorado across the 26 multispecies WGS samples. ReadChop achieved a higher mean accuracy with tighter variance compared to Dorado, highlighting ReadChop's superior precision and stability in complex real-world non-amplicon scenarios.


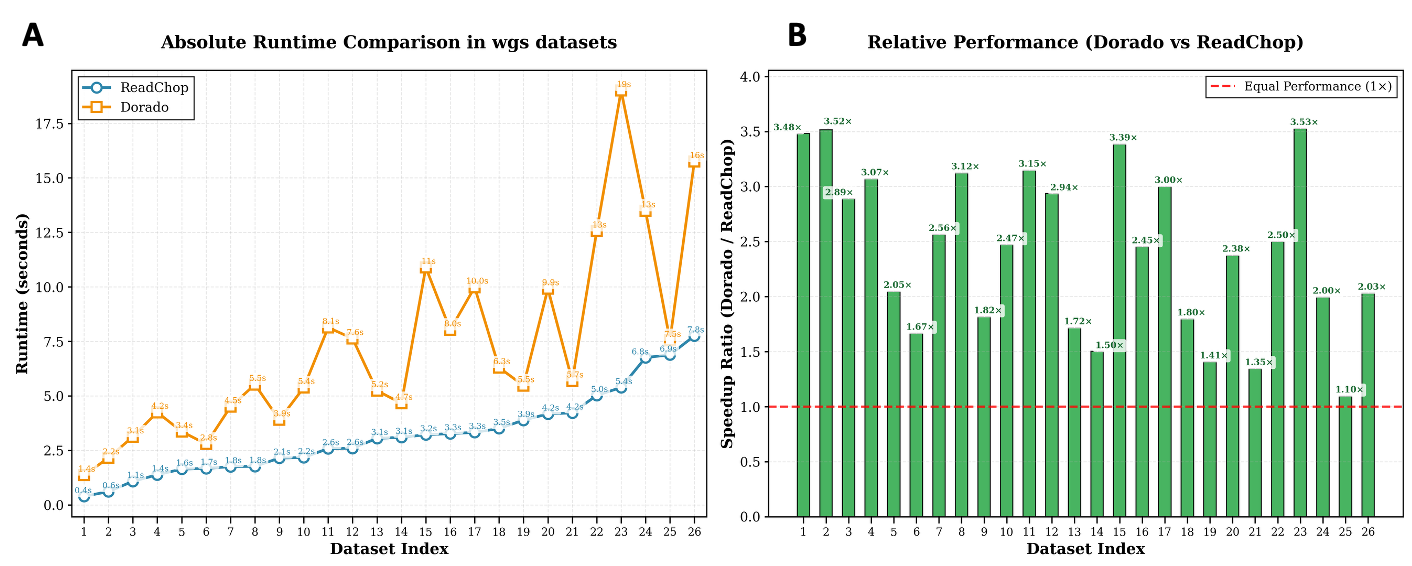


**Supplementary Figure S3. Runtime performance comparison on empirical non-amplicon datasets.** Execution speeds of ReadChop and Dorado were evaluated across 26 real multispecies Whole Genome Sequencing (WGS) samples (PRJNA1364846). (A) Absolute runtime (in seconds) for each dataset index. (B) Relative performance (speedup ratio) of ReadChop compared to Dorado. The red dashed line indicates equal performance (1x). ReadChop consistently outperformed Dorado across all empirical datasets, achieving processing speedups ranging from 1.10x up to 3.53x, demonstrating robust real-world computational efficiency.


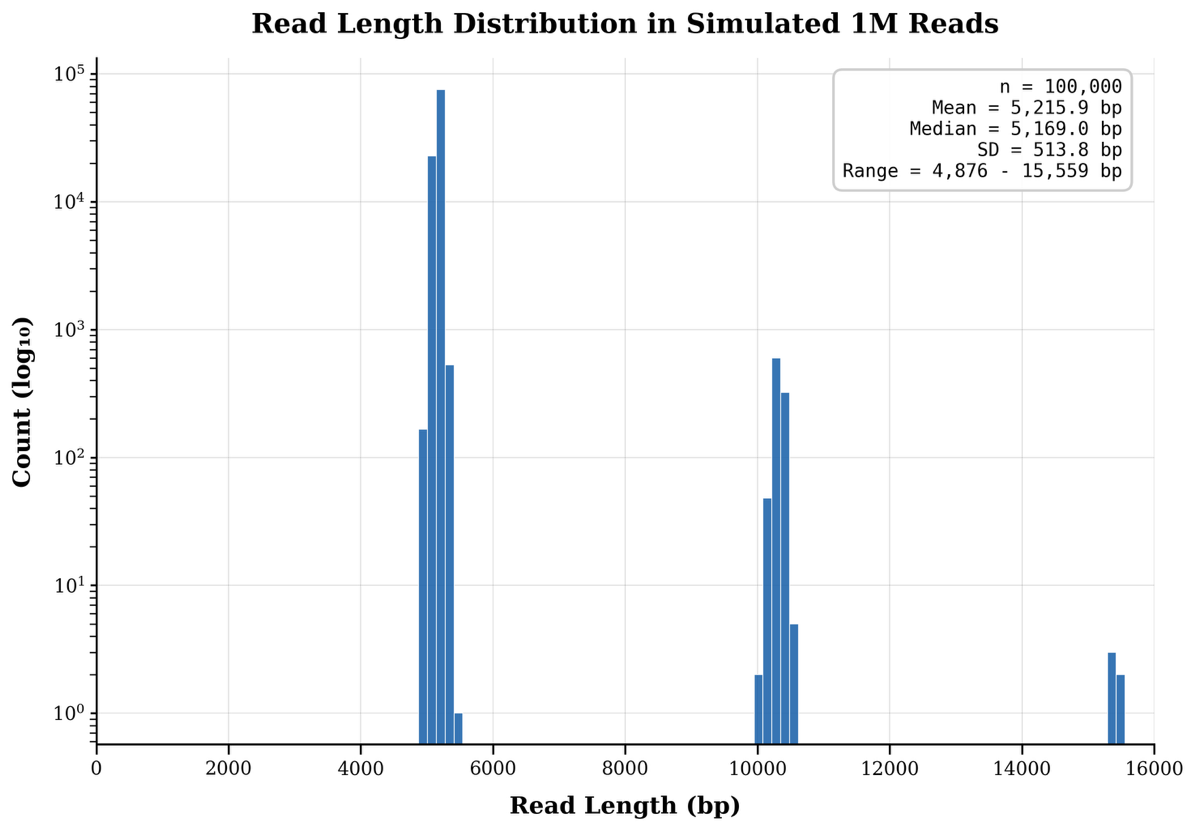


**Supplementary Figure S4. Read length distribution of the simulated dataset for chimera evaluation.** Histogram displaying the read length frequencies of 10^6^ simulated reads (target template length ~5,000 bp) with a 1% in silico chimera rate. The distinct primary peak at ~5,000 bp represents normal single fragments, while the secondary peak near 10,000 bp corresponds to concatenated chimeric reads.


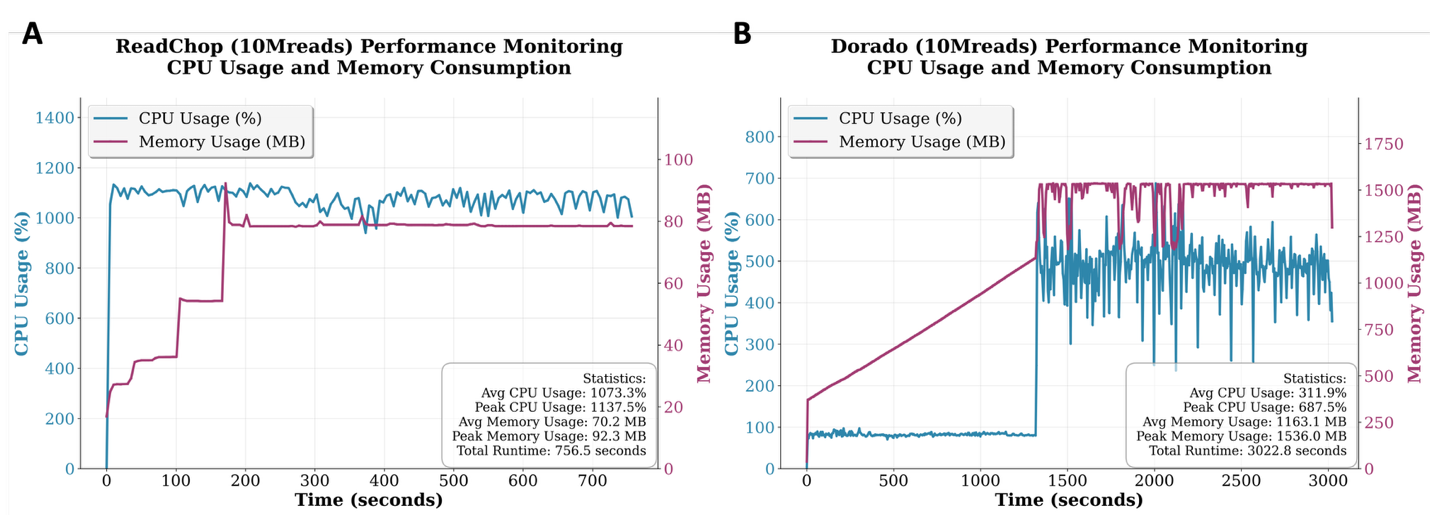


**Supplementary Figure S5. Resource utilization during stress testing on an ultra-large dataset.** Real-time monitoring of CPU usage (%) and memory consumption (MB) for processing 10^7^ reads (~100 GB of data). (A) ReadChop maintained a remarkably minimal memory footprint, peaking at only 92.3 MB with a total runtime of 756.5 seconds. (B) In contrast, Dorado required a peak memory of 1536.0 MB and took 3022.8 seconds to complete the identical task. The results underscore ReadChop's linear scalability and exceptional resource efficiency for massive sequencing initiatives.
